# Comparison of the susceptibility of *Plasmodium knowlesi* and *Plasmodium falciparum* to antimalarial agents

**DOI:** 10.1093/jac/dkx279

**Published:** 2017-08-30

**Authors:** Donelly A van Schalkwyk, Robert W Moon, Benjamin Blasco, Colin J Sutherland

**Affiliations:** 1 Department of Immunology & Infection, Faculty of Infectious & Tropical Diseases, London School of Hygiene & Tropical Medicine, Keppel Street, London WC1E 7HT, UK; 2 Medicines for Malaria Venture, 20 rte de Pré Bois, Geneva CH 1215, Switzerland; 3 Department of Clinical Parasitology, Hospital for Tropical Diseases, Mortimer Market Centre, Capper Street, London WC1E 6JB, UK

## Abstract

**Background:**

The simian malaria parasite *Plasmodium knowlesi* is now a well-recognized pathogen of humans in South-East Asia. Clinical infections appear adequately treated with existing drug regimens, but the evidence base for this practice remains weak. The availability of *P. knowlesi* cultures adapted to continuous propagation in human erythrocytes enables specific studies of *in vitro* susceptibility of the species to antimalarial agents, and could provide a surrogate system for testing investigational compounds against *Plasmodium vivax* and other non-*Plasmodium falciparum* infections that cannot currently be propagated *in vitro*.

**Objectives:**

We sought to optimize protocols for *in vitro* susceptibility testing of *P. knowlesi* and to contrast outputs with those obtained for *P. falciparum* under comparable test conditions.

**Methods:**

Growth monitoring of *P. knowlesi in vitro* was by DNA quantification using a SYBR Green fluorescent assay or by colorimetric detection of the lactate dehydrogenase enzyme. For comparison, *P. falciparum* was tested under conditions identical to those used for *P. knowlesi.*

**Results:**

The SYBR Green I assay proved the most robust format over one (27 h) or two (54 h) *P. knowlesi* life cycles. Unexpectedly, *P. knowlesi* displays significantly greater susceptibility to the dihydrofolate reductase inhibitors pyrimethamine, cycloguanil and trimethoprim than does *P. falciparum*, but is less susceptible to the selective agents blasticidin and DSM1 used in parasite transfections. Inhibitors of dihydroorotate dehydrogenase also demonstrate lower activity against *P. knowlesi*.

**Conclusions:**

The fluorescent assay system validated here identified species-specific *P. knowlesi* drug susceptibility profiles and can be used for testing investigational compounds for activity against non-*P. falciparum* malaria.

## Introduction

One of six species of *Plasmodium* that infect humans, the zoonotic parasite *Plasmodium knowlesi* is increasingly recognized as an important contributor to malaria infection in South-East Asia, including Malaysia, Myanmar and Indonesia.^1–3^ Infections are characterized by the rapid (24 h) schizogonic cycle, can be severe and are occasionally lethal. A thorough understanding of *P. knowlesi* susceptibility to both existing and pipeline antimalarial therapies is critical.

Thus far, *in vitro* screening of newly developed antimalarial drugs has been limited to *Plasmodium falciparum*—the only tractable human malaria species *in vitro* until the recent adaptation of *P. knowlesi* to continuous culture in human erythrocytes.^4–6^ Originally isolated from a Malaysian macaque in the 1960s, the culture-adapted isolate has no history of exposure to antimalarial drugs and provides an unselected genetic background on which to screen new antimalarials by assessment of parasite susceptibility *in vitro*. As early as 2004, incorporation of [^3^H]hypoxanthine was used to monitor growth of *P. knowlesi* cultured in rhesus erythrocytes following exposure to selective agents used for transfection,^7^ and in human erythrocyte-adapted *P. knowlesi* cultures.^8^*Ex vivo* drug susceptibility has been investigated using the microscopy-based WHO microtest and the colorimetric lactate dehydrogenase (LDH) assay.^9^ All studies thus far have failed to address key differences in *P. knowlesi* biology that may reduce applicability of standard assays developed for *P. falciparum*. These include albumin content of growth media, differences in life cycle length and contrasting multiplication rates. Thus meaningful, adequately controlled comparisons of *in vitro* drug susceptibility in the two parasite species have yet to be reported.

We assess the susceptibility of *P. knowlesi* cultured in human red cells against a panel of current and experimental antimalarial agents, in comparison with drug-susceptible *P. falciparum* (3D7). We evaluate assays using the DNA intercalating fluorescent dye SYBR Green I, and the LDH-based colorimetric assay, to measure parasite growth inhibition *in vitro*, and in so doing elucidate detailed susceptibility profiles for several compound classes.

## Materials and methods

### Drugs

Antimalarial compounds were provided by the Medicines for Malaria Venture, Geneva, Switzerland. Drug stocks were prepared in DMSO except chloroquine and blasticidin, which were prepared in sterile distilled water.

### Parasite culture

*P. knowlesi* (A1-H.1 clone) was cultured as described previously with minor modifications.^10^ Briefly, parasites were maintained at 2% haematocrit in RPMI 1640 supplemented with 25 mM HEPES, 25 mM Na_2_HCO_3_, 10 mM d-glucose, 2 mM l-glutamine, 25 mg/L gentamicin sulphate, 50 mg/L hypoxanthine, 5 g/L Albumax II and 10% (v/v) equine serum (Thermo Fisher Scientific, 26050-070). For routine culturing *P. falciparum* (3D7 clone) was maintained in identical growth medium, supplemented with 2% heat-inactivated human serum (Sigma–Aldrich, H4522) in place of the equine serum. For drug assays, unless stated, both parasite species were grown in the *P. knowlesi* growth medium/serum mix. Both *P. knowlesi* and *P. falciparum* parasites were grown in human A^+^ blood (National Health Blood and Transplant, UK). Some experiments were performed in blood from *Macaca fascicularis*, provided by NIBSC (UK) in K_2_EDTA vacutainers (Becton Dickinson). Parasites were incubated at 37 °C under a culture gas mixture of 93% N_2_, 4% CO_2_ and 3% O_2_.

### Synchronization

*P. knowlesi* schizont culture was adjusted to 50% haematocrit in RPMI medium; 2 mL was layered on top of 5 mL of 55% Nycodenz solution in 10 mM HEPES (pH 7.0) and centrifuged at 900 **g** for 12 min. The pigmented interphase containing mature parasites was removed and washed in RPMI then returned to culture with fresh red cells.^6^*P. falciparum* parasites were synchronized with 5% (w/v) d-sorbitol as described previously.^11^

### Growth inhibition assays

Drug susceptibility of *P. knowlesi* and *P. falciparum* was assayed using 96-well flat-bottomed microplates, with 100 μL of parasite stock added to 100 μL of drug dilution in medium per well. Drug-free control wells were included in each experiment and background fluorescence determined in parasite-seeded wells containing a supralethal concentration of chloroquine (10 μM). The plates were incubated at 37 °C in an incubation chamber (Billups-Rothenburg Inc.) under culture gas, and then stored at –20 °C overnight.

Microplates were thawed and incubated with 100 μL of SYBR Green lysis buffer [1:5000 SYBR Green I (Thermo Fisher Scientific, S7563), diluted in 20 mM Tris, 5 mM EDTA, 0.008% (w/v) saponin, 0.08% (v/v) Triton X-100, pH 7.5] in the dark for 1 h, before fluorescence was read in a Spectramax M3 microplate reader (Molecular Devices) at 490 nm excitation and 520 nm emission.

The colorimetric LDH assay was performed as described for *P. falciparum*.^12–15^ Briefly, 100 μL of LDH lysis buffer [100 mM Tris–HCl, 200 mM l-lactic acid, 0.2% (v/v) Triton X-100, 125 μM 3-acetylpyridine adenine dinucleotide], 20 μL of nitroblue tetrazolium (1.6 mg/mL) plus phenazine ethosulphate (80 μg/mL) solution and 20 μL of the resuspended parasite preparation were added to each well of a duplicate plate. The plate was developed in the dark for 30–60 min until a clear difference between drug-free controls and background controls was apparent. Parasite growth is measured by accumulation of a blue formazan salt, giving absorbance at 650 nm.^9^^,^^14^^,^^16^

### Time course

To test for the effect of parasite synchrony on drug responses, we initiated a time course of drug susceptibility assays at 6 or 12 h intervals across the *P. knowlesi* and *P. falciparum* life cycles of 27 and 48 h, respectively. Late-stage parasites were synchronized with a 2 h window using sequential Nycodenz purification as described previously.^6^ New ring stages (0–2 h post-invasion) were diluted to 1% parasitaemia and exposed to drugs (as described above) for one or two life cycles (27 or 54 h for *P. knowlesi* A1-H.1^6^ and 48 or 96 h for *P. falciparum* 3D7). From this parasite stock, subsequent drug assays on *P. knowlesi* were initiated every 6 h for 24 h and on *P. falciparum* every 12 h for 36 h.

### Statistics

*Z*′ factors were calculated to measure the assay quality as described previously,^17^ using assay plates containing six negative control wells and six positive control wells. Assays with *Z*′ values lying between 0.5 and 1.0 are considered indicative of a robust assay performance. *P* values were calculated using Student’s two-tailed *t*-test for unpaired or paired samples.

## Results and discussion

### Effect of starting parasitaemia and haematocrit on non-isotopic growth assays

Although previously used for parasite growth assay in *P. knowlesi*,^7^^,^^8^^,^^18^ the requirement for radiolabelled hypoxanthine and specialized equipment prevent the [^3^H]hypoxanthine incorporation assay from being widely implemented. We therefore focused our attention on optimization of two non-isotopic methods, namely the fluorometric SYBR Green I assay and the colorimetric LDH enzyme assay, to measure and compare *in vitro* drug susceptibility between *P. knowlesi* and *P. falciparum*.

**Figure 1.**
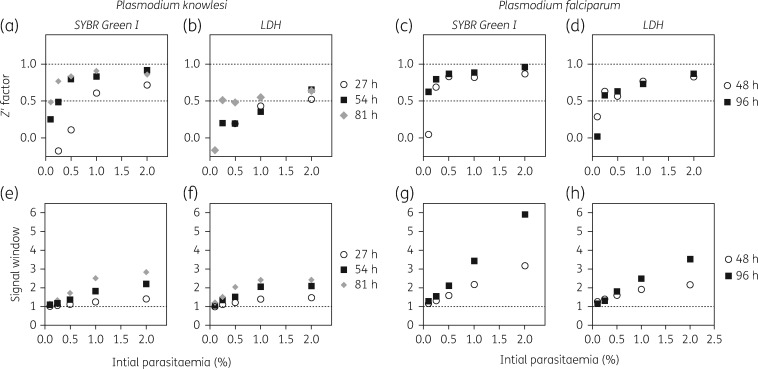
Influence of starting parasitaemia of *P. knowlesi* (A1-H.1) and *P. falciparum* (3D7) on assay quality for both the fluorescent and colorimetric methods. Parasites set to 1% haematocrit and varying parasitaemia (0.1%–2%) were cultured in the presence or absence of a supralethal concentration of chloroquine for 27 h (circles), 54 h (squares) or 81 h (diamonds) for *P. knowlesi*, and 48 h (cirlces) or 96 h (squares) for *P. falciparum*. Upon termination of the assay, the plates were read using either the SYBR Green I fluorescence assay (a, c, e and g) or the LDH assay (b, d, f and h). The signal window and *Z*′ factor were calculated for each assay. The signal window was calculated by dividing the average reading for the drug-free control by the average reading for the high chloroquine concentration (background) control. The assay quality was assessed by determining the *Z*′ factor using the formula described in Zhang *et al.*^17^

*P. knowlesi* and *P. falciparum* parasites were diluted to a series of starting parasitaemia at 1% haematocrit (Figure 1) or 2% haematocrit (Figure S1, available as Supplementary data at *JAC* Online). Whilst the *P. knowlesi* life cycle *in vivo* is 24 h, the life cycle *in vitro* takes longer at 27 h, and incubation times were modified accordingly. Cultures were therefore incubated in the presence or absence of drugs for one, two or three complete life cycles: 27, 54 and 81 h for *P. knowlesi*; 48 and 96 h for *P. falciparum*.

For *P. knowlesi*, the SYBR Green I assay produced high-quality results for a single life cycle exposure (27 h) using a starting parasitaemia of 1% and 1% haematocrit (Figure 1a). Lower starting parasitaemia also generated good-quality assays if exposed for two (54 h) or three (81 h) life cycles. LDH assays starting at 1% parasitaemia/1% haematocrit yielded assays of only borderline quality and parasitaemia below 1% gave unsatisfactory results—thus initiating assays at 2% parasitaemia is preferable for this method (Figure 1b). The signal window improved with longer exposures at all starting parasitaemia for the SYBR Green assay (Figure 1e) and the LDH assay (Figure 1f) but remained <3.0 for both assay methods.

For *P. falciparum*, good-quality assays were obtained at starting parasitaemia of 0.25% and 1% haematocrit for a single life cycle (48 h) and at 0.1% parasitaemia/1% haematocrit for two life cycles (96 h) using the SYBR Green I method (Figure 1c). Similarly, the LDH assay performed better with *P. falciparum* down to 0.25% starting parasitaemia (Figure 1d). Again, the signal window improved with higher initial parasitaemia and with longer exposures for both the SYBR Green (Figure 1g) and LDH assays (Figure 1h). The species-specific difference in assay quality for both formats is partly explained by the lower multiplication rate per life cycle for *P. knowlesi* (3- to 4-fold) compared with *P. falciparum* (6- to 8-fold). Furthermore, the activity of the LDH enzyme is poorly characterized in *P. knowlesi* relative to *P. falciparum*.

### Effect of synchrony on drug susceptibility measured across the life cycle

**Figure 2.**
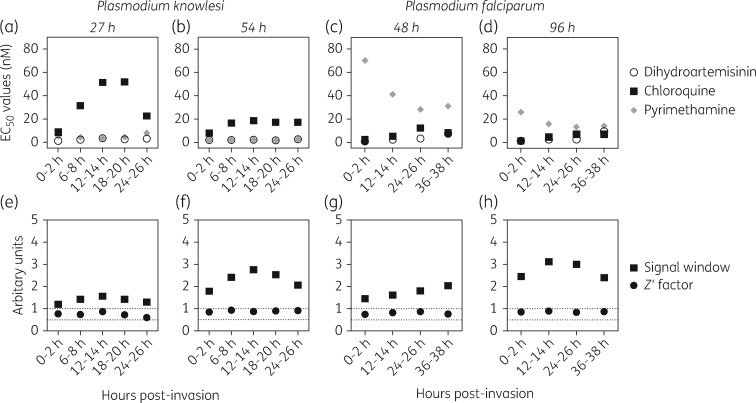
Effect of synchrony on drug susceptibility measured across the life cycle using the SYBR Green I method after one or two life cycles. EC_50_ values for chloroquine (squares), dihydroartemisinin (circles) or pyrimethamine (diamonds) were determined from experiments initiated at the times shown on the *y*-axis using synchronized parasites at 1% parasitemia and 1% haematocrit that were incubated for either 27 h (a) or 54 h (b) for *P. knowlesi*, and 48 h (c) or 96 h (d) for *P. falciparum*. Signal window (squares) and assay quality (*Z*′ factor, circles) (e–h) were determined as in Figure 1.

Drug susceptibility testing of *P. falciparum* is usually initiated using sorbitol-synchronized ring-stage parasites. *P. knowlesi* is less amenable to sorbitol synchronization, requiring density gradient synchronization instead, and also loses synchrony rapidly *in vitro*. To examine the effect of synchrony on susceptibility to antimalarial agents, a time course was initiated with synchronized *P. knowlesi* or *P. falciparum* exposed to chloroquine, dihydroartemisinin or pyrimethamine for one and two complete life cycles, and results compared between the SYBR Green I fluorescence method (Figure 2) and the colorimetric method (Figure S2).

In the fluorescence assay after one cycle with *P. knowlesi*, the initial life cycle stage had little effect on the EC_50_ for either the endoperoxide dihydroartemisinin or the antifolate pyrimethamine, but the EC_50_ varied dramatically with chloroquine (Figure 2a). For *P. falciparum* there was relatively little variability in EC_50_ values for chloroquine and dihydroartemisinin but large differences for pyrimethamine after a single 48 h exposure (Figure 2c). For both species, variability between EC_50_ values at different initial life cycle stages was markedly reduced when samples were read after two cycles (Figure 2b and d).

The fluorescence method yielded good *Z*′ factors of between 0.6 and 0.91 for *P. knowlesi* (Figure 2e and f) and between 0.75 and 0.89 for *P. falciparum* (Figure 2g and h), supporting the use of the SYBR Green I method for assays initiated at 1% parasitaemia and 1% haematocrit on parasites of varying synchrony. For both species, timing of initiation of the experiment and use of double life cycle exposure were important determinants of quality (Figure 2f versus e and Figure 2h versus g).

Synchronized assays read by the LDH method (Figure S2) showed a similar pattern to those read for the fluorescence assay after one cycle. Highly variable EC_50_ estimates were obtained with chloroquine in *P. knowlesi*, and pyrimethamine in *P. falciparum*, depending on the initial life cycle stage. For *P. knowlesi*, EC_50_ values could not be obtained for pyrimethamine in 27 h experiments initiated in early trophozoites (Figure S2A), even though estimates were readily obtained using the SYBR Green I method (Figure 2a). This suggests that short exposures of pyrimethamine were able to inhibit DNA replication but not LDH activity in *P. knowlesi*. By the second life cycle exposure LDH activity was inhibited at higher drug concentrations and all assays yielded EC_50_ values (Figure S2B). Similarly, one of the *P. falciparum* curves for pyrimethamine failed to yield an EC_50_ estimate after 48 h exposure but was able to generate data after 96 h exposure. This is clearly a weakness of enzyme-based assays for measuring parasite growth and may lead to otherwise active, potent compounds being incorrectly rejected if the timing or duration of exposure is non-optimal. As in the previous assays, two-cycle experiments greatly reduced any variation in EC_50_ caused by altering the initial life cycle stage.

Whilst the LDH assays performed on *P. falciparum* were of good quality (Figure S2G and H), the assays on *P. knowlesi* performed poorly with a small signal window and low *Z*′ factor after a single life cycle (<2; Figure S2E). Signal and assay quality improved with double life cycle exposure (*Z*′ range = 0.51–0.77; Figure S2F). This suggests that, for *P. knowlesi*, the LDH assay is not ideal for short-exposure drug assays initiated at 1% parasitaemia and 1% haematocrit. The LDH assay is suitable for *P. falciparum* drug assays but caution is needed when examining the effect of antifolates such as pyrimethamine. Although EC_50_ results for synchronous single-cycle experiments varied dramatically depending on the initial life cycle stage in *P. knowlesi* (Figure 2a), the mean EC_50_ obtained from these synchronized assay data closely approximated the EC_50_ estimates from experiments on non-synchronous parasites (Figure S3). Thus, in addition to being logistically simpler, the non-synchronous experiments can ameliorate the variation observed due to stage-specific effects in synchronous experiments. Considering this, and the variable performance of the LDH platform, all subsequent susceptibility testing in *P. knowlesi* deployed the non-synchronous fluorescent SYBR Green I method.

### Activity of antimalarial agents

Using starting conditions of 1% parasitaemia and 1% haematocrit, we compared the drug susceptibility of *P. knowlesi* and *P. falciparum* (3D7) exposed for one complete life cycle. As *P. knowlesi* requires media heavily supplemented with Albumax/serum, all EC_50_ experiments were carried out in the *P. knowlesi* media, which readily supports growth of both parasite species. This removes the confounding effect of serum protein levels on EC_50_ estimates for certain drugs (e.g. atovaquone; Table S1).

The susceptibility of *P. knowlesi* to the 4-aminoquinolines and amino-alcohols was similar to that of *P. falciparum* (Table 1). All EC_50_ estimates for *P. knowlesi* fell below 100 nM and within 2.5-fold of the EC_50_ reported for *P. falciparum* (Table 1). Although the EC_50_ differences were not large between species, several were statistically significant (*P* ≤ 0.0424). Ferroquine, currently in Phase II trials, was highly potent against *P. knowlesi* (12.2 nM; Table 1).

**Table 1.** Comparison of the antiplasmodial activity against *P. knowlesi* or *P. falciparum*, assessed using the SYBR Green I assay, for a set of clinical and experimental antimalarials exposed over one complete life cycle

Compound	EC_50_ values (nM)		Fold difference (*P. falciparum*/*P. knowlesi*)	*P*^a^
	*P. knowlesi* (A1-H.1), 27 h exposure	*P. falciparum* (3D7), 48 h exposure		
4-Aminoquinolines and amino-alcohols				
chloroquine	29.3 ± 4.7	15.9 ± 3.0	0.54	0.0303
amodiaquine	9.3 ± 1.7	5.9 ± 0.6	0.63	0.0662
desethylamodiaquine	12.4 ± 1.4	12.4 ± 3.1	1.00	0.9973
quinine	54.8 ± 3.0	57.9 ± 6.9	1.06	0.7177
mefloquine	10.9 ± 1.1	26.2 ± 4.2	2.40	0.0090
lumefantrine	90.4 ± 13	152 ± 26	1.68	0.0424
piperaquine	21.0 ± 3.1	39.8 ± 4.9	1.90	0.0115
pyronaridine	10.7 ± 1.6	4.4 ± 1.6	0.41	0.0268
ferroquine^b^	12.2 ± 1.6	4.7 ± 0.6	0.39	0.0068
Endoperoxides				
dihydroartemisinin	2.0 ± 0.3	4.2 ± 0.5	2.10	0.0098
artesunate	10.9 ± 1.7	9.0 ± 1.5	0.83	0.4280
OZ439^b^	6.6 ± 1.4	7.4 ± 1.2	1.12	0.6750
DHFR inhibitors				
pyrimethamine	5.1 ± 0.8	54.0 ± 5.0	10.6	<0.0001
cycloguanil	1.3 ± 0.3	11.8 ± 0.6	9.08	<0.0001
trimethoprim	265±47	3098 ± 229	11.7	<0.0001
P218^b^	4.1±0.7	3.5 ± 0.2	0.85	0.4884
Transfection reagents				
WR99210^c^	0.16 ± 0.04	0.43 ± 0.03	2.69	0.0003
blasticidin^c^	31684 ± 3485	1413 ± 190	0.04	<0.0001
DSM1^c^	509 ± 11	149 ± 5	0.29	<0.0001
Other				
primaquine	3871 ± 887	5627 ± 1195	1.45	0.2847
atovaquone	2.6 ± 0.4	2.3 ± 0.5	0.88	0.6366

EC_50_ values are reported as mean ± SEM from at least three experiments, and some up to eight repeats, each performed in duplicate.

a *P* values are calculated by comparing EC_50_ values for *P. falciparum* versus *P. knowlesi* using Student’s two-tailed unpaired *t*-test.

b These agents are undergoing development for their potential use as antimalarial agents (http://www.mmv.org/research-development/interactive-rd-portfolio).

c These compounds are used in transfection studies with *P. falciparum* to select for parasites that harbour plasmids carrying drug resistance cassettes.

Presently, artemisinin-based combination therapy is recommended for the treatment of uncomplicated *P. knowlesi* malaria.^19^ Artesunate, dihydroartemisinin and a synthetic endoperoxide, OZ439, were all highly potent against both parasite species, with *P. knowlesi* significantly more susceptible to dihydroartemisinin than *P. falciparum* (Table 1; *P *=* *0.0098).

Interestingly, we found *P. knowlesi* parasites to be highly susceptible to dihydrofolate reductase (DHFR) inhibitors, being more than 9-fold more susceptible to pyrimethamine, cycloguanil and trimethoprim than the drug-susceptible *P. falciparum* line tested here. However, both species showed similar susceptibility (∼4 nM) to the new DHFR inhibitor P218, designed to overcome resistant forms of the *P. falciparum* enzyme.^20^ Thus existing medicines such as sulfadoxine/pyrimethamine may prove to be very effective agents against *P. knowlesi*, both for treatment and prophylaxis. Future studies should explore the impact of both DHFR and dihydropteroate synthase inhibitors on *P. knowlesi* metabolism in depth, the latter requiring specialized growth media sufficiently depleted of folate and *para*-aminobenzoic acid,^21^^,^^22^ and so not tested here.

### Transfection reagents

We tested *P. knowlesi* susceptibility to three common selective agents used to favour growth of transfected *P. falciparum* parasites harbouring exogenous DNA. The DHFR inhibitor WR99210 was highly potent against *P. knowlesi* with an EC_50_ value of 0.16 ± 0.04 nM. Similar to other DHFR inhibitors tested, WR99210 was significantly more potent against *P. knowlesi* than against *P. falciparum* (0.43 ± 0.03 nM; *P *=* *0.0003). Blasticidin was 22-fold less potent against *P. knowlesi* when compared with *P. falciparum* (Table 1) over a single life cycle, which is consistent with a previous report,^7^ in which *P. knowlesi* H strain was grown in rhesus erythrocytes. Reduced susceptibility of *P. knowlesi* to blasticidin prevents its use as a selectable marker at the concentrations generally used for transfection studies. Similarly, *P. knowlesi* was also 3-fold less susceptible than 3D7 to DSM1 (Table 1); dihydroorotate dehydrogenase (DHODH)-containing plasmid selection with DSM1 needs to be conducted at higher concentrations for this species.

*P. knowlesi* and *P. falciparum* were both highly susceptible to the mitochondrial cytochrome *b* inhibitor atovaquone (Table 1), but both poorly susceptible to primaquine *in vitro* with EC_50_ values at micromolar concentrations.

### DHODH inhibitors

The DHODH enzyme is a newly validated antimalarial target.^18^^,^^23–25^ Several inhibitors of this enzyme have been identified and the two most advanced, DSM421 and DSM265, are currently in preclinical and Phase II trials.^23^^,^^25^ Considering the reduced potency of the transfection reagent DSM1 against *P. knowlesi* versus *P. falciparum*, we tested other DHODH inhibitors against *P. knowlesi* (Table 2). All compounds were ≥2.7-fold more potent against *P. falciparum* than against *P. knowlesi* whether tested over one or two life cycles. In particular, DSM265 was 8-fold more potent against *P. falciparum* (Table 2). This supports previous observations suggesting that *P. knowlesi* was less susceptible to DHODH inhibitors than *P. falciparum*.^18^ However, in that study the two species were tested under completely discordant assay conditions. Whether *P. knowlesi* is less susceptible than *P. falciparum* to the effects of DHODH inhibitors because of differences in enzyme activity, access to the enzyme or fundamental differences in the biology of these parasites remains to be established. Of note, DSM265 was shown to be about 5-fold more active against *P. falciparum* field isolates than *Plasmodium vivax* field isolates *ex vivo*, while DSM421 was equipotent against both species,^25^ reinforcing the relevance of using *P. knowlesi* drug susceptibility testing to inform drug development for *P. vivax*.^4^

**Table 2.** Susceptibility of *P. knowlesi* and *P. falciparum* to three DHODH inhibitors assessed using the SYBR Green I assay

DHODH inhibitor	EC_50_ (nM), single life cycle			EC_50_ (nM), two life cycles		
	*P. knowlesi*, 27 h	*P. falciparum*, 48 h	fold difference	*P. knowlesi*, 54 h	*P. falciparum*, 96 h	fold difference
DSM1	509 ± 11	149 ± 5	3.4	417 ± 2	91 ± 10	4.6
DSM265	303 ± 15	37 ± 3	8.2	186 ± 11	21 ± 1	8.9
DSM421	194 ± 23	72 ± 5	2.7	123 ± 10	42 ± 4	2.9

All inhibitors were tested in duplicate on three separate occasions from a starting parasitaemia and haematocrit of 1%. The EC_50_ values are reported as the mean ± SEM. The fold difference is calculated by dividing the *P. knowlesi* EC_50_ value by the *P. falciparum* EC_50_ value. For each DSM compound, the mean *P. falciparum* EC_50_ value was significantly lower than the mean *P. knowlesi* EC_50_ value when compared over either a single parasite life cycle or over two life cycles (*P *≤* *0.0018).

### Delayed death effect

Antibacterial agents, such as azithromycin and clindamycin, have been shown to exert potent activity against *P. falciparum in vitro* but only after two complete asexual life cycles (96 h).^26^ This phenomenon is referred to as the delayed death effect, and has also been reported for clindamycin against *P. knowlesi in vitro*.^8^ In our experiments, a delayed death effect in *P. knowlesi* is confirmed for clindamycin, doxycycline and azithromycin (Table 3). EC_50_ values for *P. knowlesi* were measured over three life cycles (81 h), as additional time was required to resolve the full delayed drug effect in our experiments using unsynchronized cultures. For *P. falciparum* parasites the assay used synchronized parasites, and therefore, two cycles (96 h) were sufficient to detect the delayed death effect. Azithromycin was equally potent between species over a single life cycle (*P *=* *0.4397) and not significantly different in its delayed death effect (*P *=* *0.2514). Similarly, clindamycin had no measurable effect over a single life cycle in either species but was very potent against *P. knowlesi* (15.9 nM) and *P. falciparum* (7.0 nM) over 81 and 96 h, respectively. For doxycycline the delayed death potency for *P. knowlesi* was much reduced (2061 nM) relative to *P. falciparum* (623 nM). We noted that the delayed death curves did not level out to 0% viability but were asymptotic at about 25% viability, presumably due to the greater amount of residual DNA from parasites surviving the first cycle of growth compared with the chloroquine control wells in which parasites die in the first cycle. This was corrected for clindamycin and doxycycline, but not azithromycin, by using a background control generated for the second cycle only (Figure S4).

**Table 3.** Delayed death effect of three antibacterial agents against *P. knowlesi* and *P. falciparum* assessed using the SYBR Green I assay

Antibacterial	*P. knowlesi* EC_50_ (nM)			*P. falciparum* EC_50_ (nM)		
	27 h	81 h	fold difference	48 h	96 h	fold difference
Azithromycin	5662 ± 725	31.9 ± 10	177	6003 ± 323	19.2 ± 4	313
Doxycycline	>10000	2061 ± 343	>4.9	>10000	623 ± 148	>16
Clindamycin	>10000	15.9 ± 4	>629	>10000	7.0 ± 1.0	>1429

All antibacterial agents were screened in duplicate on at least three separate occasions from a starting parasitaemia and haematocrit of 1%. The EC_50_ values are reported as the mean ± SEM. The fold difference for each compound is calculated by dividing the EC_50_ value after three life cycles (for *P. knowlesi*) or two life cycles (for *P. falciparum*) by the EC_50_ value measured after a single life cycle exposure.

### Drug susceptibility of P. knowlesi grown in human versus macaque blood

We assessed the effect of culturing *P. knowlesi* parasites in human versus macaque erythrocytes on susceptibility to a subset of antimalarials (Table 4). No significant host-specific differences in potency were observed, although it was evident that the EC_50_ values were generally higher in parasites grown in macaque erythrocytes (Table 4). This could be related to higher growth rates of *P. knowlesi* parasites in macaque cells, estimated at 5- to 7-fold compared with 3- to 4-fold in human erythrocytes.^6^

**Table 4.** Comparison of the susceptibility of *P. knowlesi* grown in either human or macaque red blood cells to selected antimalarial agents assessed using the SYBR Green I assay

Antimalarial	EC_50_ (nM)		Fold difference	*P*
	human blood	macaque blood		
Chloroquine	24.6 ± 3.3	42.0 ± 8.1	0.59	0.2114
Dihydroartemisinin	1.9 ± 0.2	4.1 ± 0.7	0.46	0.1250
Quinine	48.0 ± 6.7	54.7 ± 1.4	0.88	0.4303
Mefloquine	11.0 ± 1.5	18.3 ± 0.2	0.60	0.0502
Pyrimethamine	8.9 ± 0.8	13.2 ± 2.7	0.67	0.2940

Antimalarial agents were screened in duplicate on three separate occasions from a starting parasitaemia and haematocrit of 1%. The EC_50_ values are reported as the mean ± SEM. The fold difference for each compound is calculated by dividing the EC_50_ in human blood by the EC_50_ value in macaque blood measured after a single life cycle exposure (27 h).

The *P. knowlesi* A1-H.1 is descended from a 1964 macaque isolate and is assumed to be drug susceptible.^27^^,^^28^ Using identical growth media and viability readouts, we expected to find EC_50_ estimates very similar to those for *P. falciparum* 3D7 for most, if not all, antimalarials tested. The unexpected differences in susceptibility to DHFR inhibitors (pyrimethamine, cycloguanil and trimethoprim) and DHODH inhibitors suggest that important species-specific differences in drug responses exist. A recent study reported the *in vitro* activity of the 400 compound Malaria Box against *P. falciparum* 3D7 and showed that 90% were also active against *P. knowlesi* yH-1 strain.^29^ Closer examination of those data show that EC_50_ estimates for 52 compounds were at least 3-fold higher or lower for *P. knowlesi* than for *P. falciparum*.

### Conclusions

We have provided detailed validation of a fluorescent assay system for drug susceptibility testing in *P. knowlesi.* This provides an important new tool for *in vitro* drug studies in non-*P. falciparum* malaria. Significant species-specific differences in susceptibility to certain compound classes was observed, highlighting the added value of *in vitro* screens against additional human malaria pathogens. The generalizability of our findings should now be tested in recent *P. knowlesi* field isolates from geographically distinct regions of South-East Asia.

## Supplementary Material

Supplementary Data
