# Supplementary material for: Comparison of the susceptibility of Plasmodium knowlesi and Plasmodium falciparum to antimalarial agents
Source: J Antimicrob Chemother. 2017 Aug 30;72(11):3051–8. doi: 10.1093/jac/dkx279 (PMC5890772; doi:10.1093/jac/dkx279)
Supplement: Supplementary Data [file dkx279_supplementary_data.docx]

**Supplementary data**

**Figure S1.** Influence of starting parasitaemia of *P. knowlesi* (A1-H.1) and *P. falciparum* (3D7) on the assay signal window and Z´ factor for both the fluorescent SYBR Green I assay and colourimetric parasite lactate dehydrogenase (pLDH) assay. Parasites set to 2% haematocrit and varying parasitaemia (0.1% to 2%) were cultured in the presence or absence of a supralethal concentration of chloroquine for 27 hours (circles), 54 hours (squares) or 81 hours (diamonds) for *P. knowlesi* or 48 hours (circles) and 96 hours (squares) for *P. falciparum*. Upon termination of the assay, the plates were read using either the SYBR Green I fluorescence assay (A, C, E and G) or the pLDH assay (B, D, F and H). Thereafter, the signal window and Z´ factor was calculated for each assay. The signal window was calculated by dividing the average reading for the drug-free control by the average reading for the high chloroquine concentration (background) control. The Z´ factor was calculated using the method of Zhang et al, 1999. ^17^

**
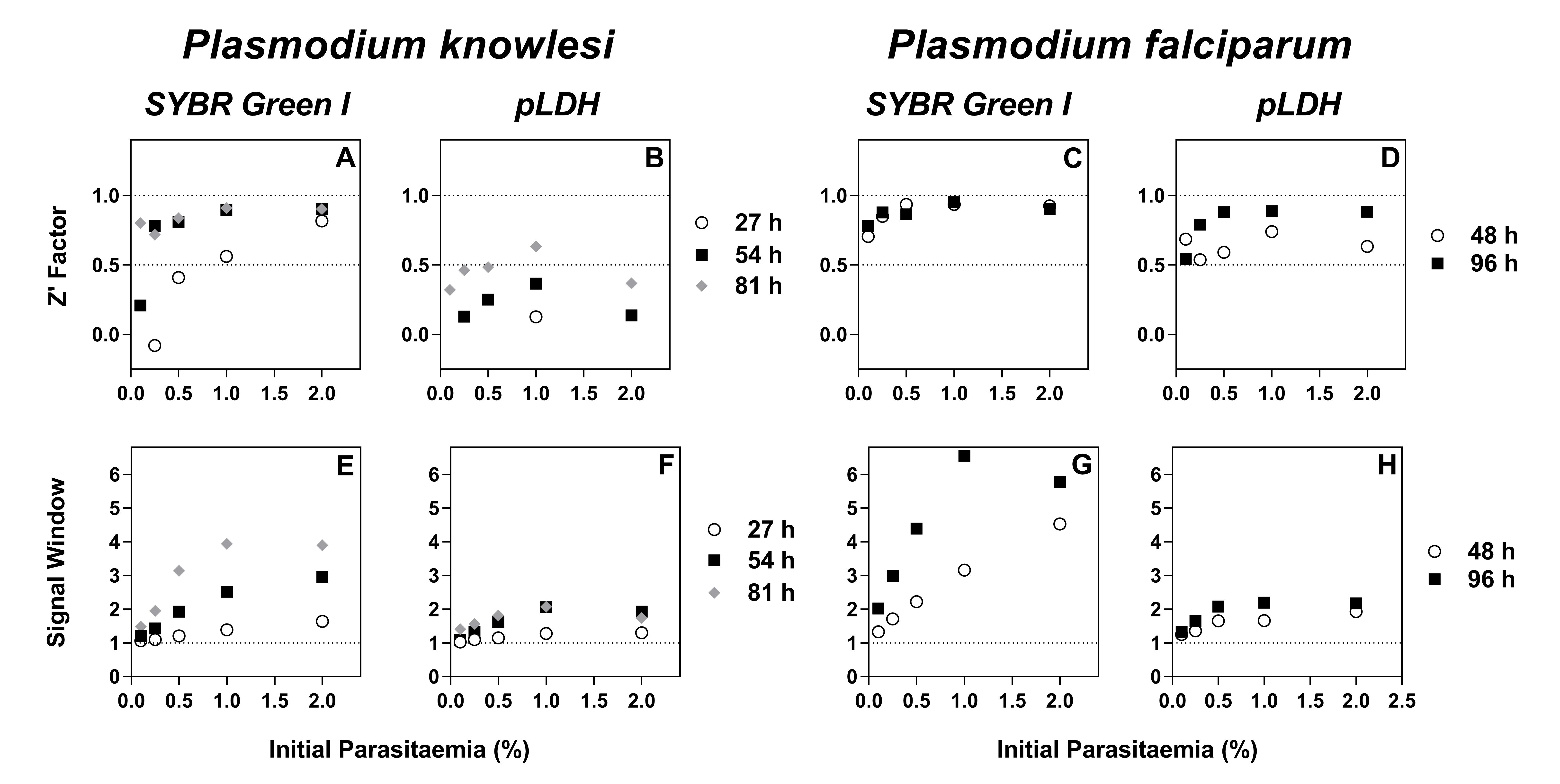
**

**Figure S2.** Effect of synchrony on drug susceptibility measured across the life cycle measured using the pLDH colourimetric method after one or two life cycles. EC_50_ values for chloroquine (squares), dihydroartemisinin (circles) or pyrimethamine (diamonds) were determined from experiments initiated at the times shown on the x-axis using synchronised *P. knowlesi* and *P. falciparum* as in Fig. 2 (A-D). Assay quality was determined as in Fig.1 (E – H).


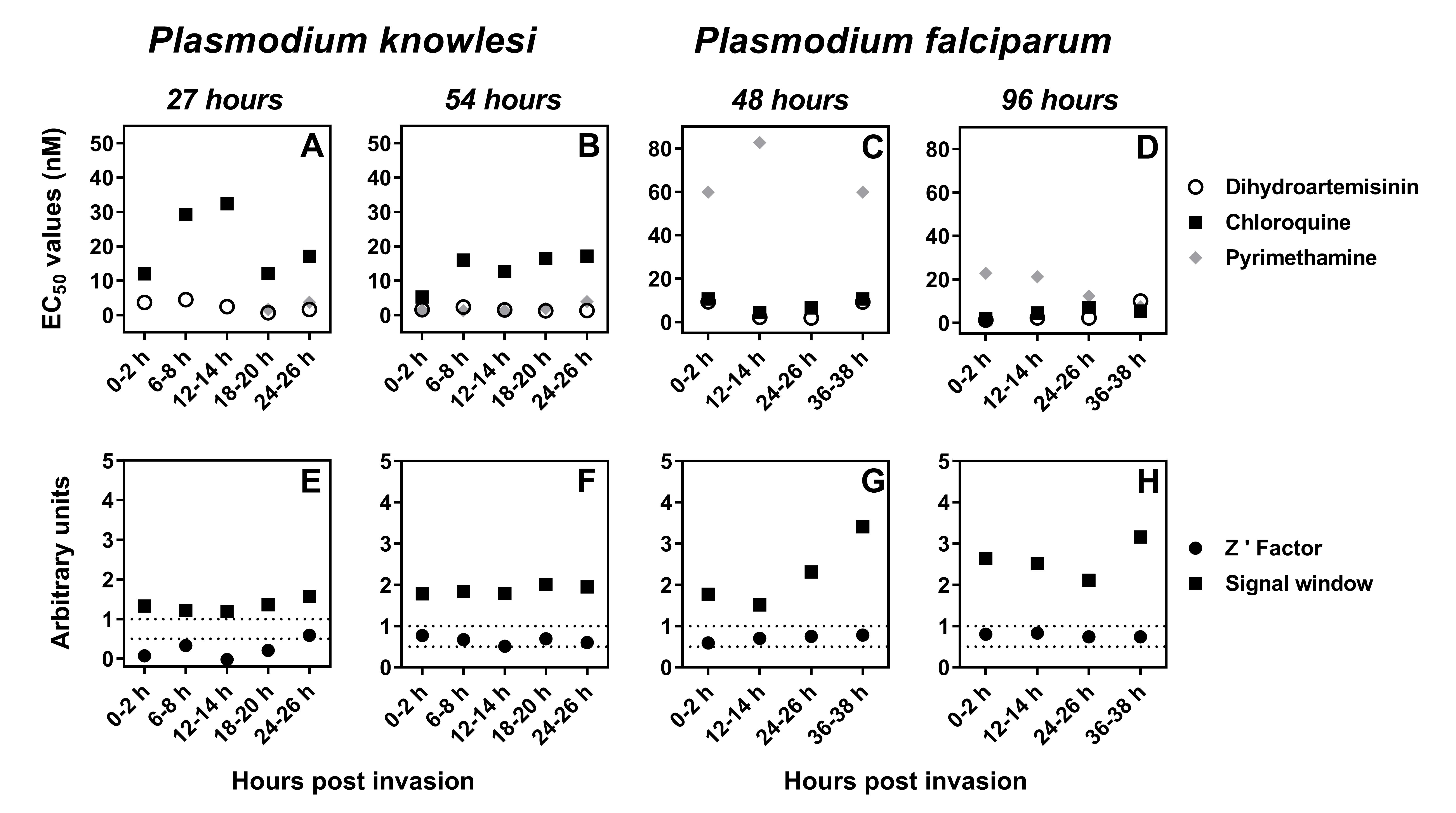


**Figure S3.** Comparison of the EC_50_ values for *P. knowlesi* determined from assays initiated using tightly synchronous cultures (symbols) at various stages of the life cycle with that of asynchronous cultures (dotted line). EC_50_ data from Figure 2A (synchronous parasites) were merged with mean EC_50_ values reported in Table 1 (asynchronous parasites). The EC_50_ data in Figure 2A for chloroquine (range 8.9-51.6 nM; mean 33.2 nM), dihydroartemisinin (range 1.2-3.5 nM; mean 2.5 nM) and pyrimethamine (range 3.5-7.9; mean 5.1 nM) were similar to the EC_50_ values reported in Table 1 for asynchronous cultures (values reported inside panels above or below dotted lines). The assay conditions were identical with respect to starting parasitaemia (1%), haematocrit (1%) and assay length (27 h) and were all read using the SYBR green I fluorescence assay.


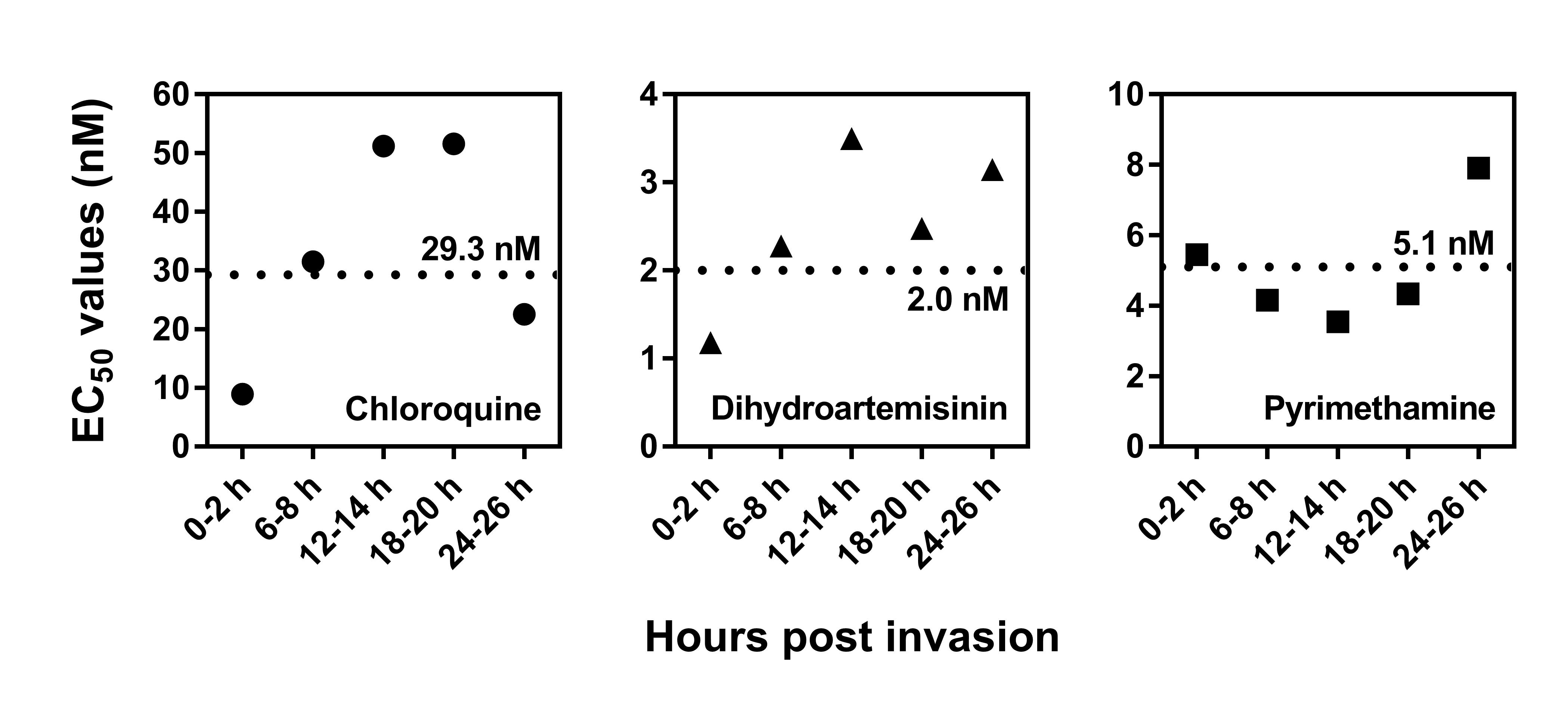


**Figure S4.** Effect of timing of the background control on the curves showing the delayed death effect using the SYBR Green I method. For the background control a supralethal concentration of chloroquine (10 µM) was added either at the start of the assay (0 h, squares) or after 54 h (circles) for *P. knowlesi* and 48 h (circles) for *P. falciparum*. The assays were terminated at 81 h for *P. knowlesi* and at 96 h for *P. falciparum*. For clindamycin, subtracting the background control prepared at the start of the assay (t = 0 h) consistently resulted in the curves not levelling out below 25% viability (A and B). Subtracting the controls prepared after the first life cycle resulted in the parasite viability dropping to 0% for both *P. knowlesi* (A) and *P. falciparum* (B). However, for azithromycin the curves never dropped to 0% viability irrespective of when the background control was prepared for *P. knowlesi* (C) and *P. falciparum* (D). Of note, the EC_50_ values calculated for each pair of curves were almost identical irrespective of where the curve levelled out.


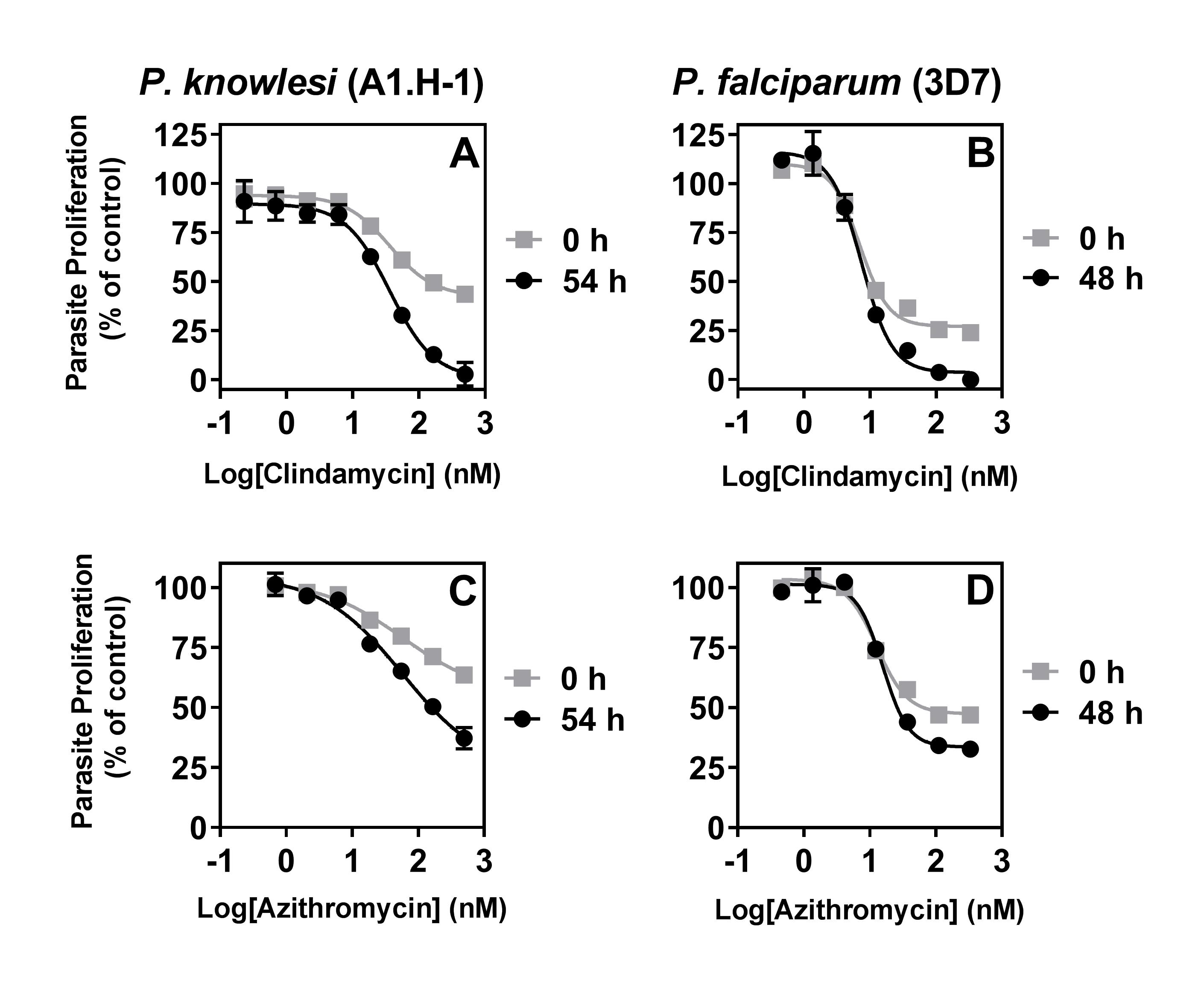


**Table S1.** High serum *P. knowlesi* growth media decreases the susceptibility of *P. falciparum* clone 3D7 to atovaquone almost 10-fold

| Media composition | Atovaquone EC_50_ (nM)* |
| --- | --- |
| RPMI + Albumax / no horse serum | 0.3 ± 0.2 |
| RPMI + Albumax + 10% horse serum | 2.6 ± 0.4 |

* EC_50_ values are averaged from at least 3 experiments each performed in duplicate. All data are presented as mean ± S.E.M.
